# Global Research Trends on Endothelial Glycocalyx in Sepsis: A Bibliometric Analysis

**DOI:** 10.1155/bmri/9720166

**Published:** 2026-03-13

**Authors:** Kamila R. Daniyarova, Zhanslu N. Sarkulova, Amin Tamadon, Ainur B. Tokshilykova, Marat N. Sarkulov, Botagoz M. Kalieva, Nadiar M. Mussin, Ramazon Safarzoda Sharoffidin

**Affiliations:** ^1^ Department of Anesthesiology and Reanimatology, West Kazakhstan Marat Ospanov Medical University, Aktobe, Kazakhstan; ^2^ Department of Natural Sciences, West Kazakhstan Marat Ospanov Medical University, Aktobe, Kazakhstan; ^3^ Department of Surgical Diseases No.2 With a Course in Urology, West Kazakhstan Marat Ospanov Medical University, Aktobe, Kazakhstan; ^4^ Department of Surgery No. 2, West Kazakhstan Marat Ospanov Medical University, Aktobe, Kazakhstan; ^5^ Department of Pharmaceutical Technology, Avicenna Tajik State Medical University, Dushanbe, Tajikistan, tajmedun.tj

**Keywords:** bibliometrics, glycocalyx, microcirculation, sepsis, syndecan-1

## Abstract

**Background:**

Sepsis remains a major global health challenge, with glycocalyx dysfunction playing an important role in its pathogenesis. Recent research highlights EG degradation as a key contributor to vascular permeability, inflammation, and organ failure. Despite growing interest, a comprehensive bibliometric analysis of global research trends on EG in sepsis is lacking.

**Methods:**

We conducted a bibliometric analysis using data from Web of Science and Scopus (2005–2025). Inclusion criteria were original English‐language research articles. Bibliometrix R‐package was employed to analyze publication trends, citation metrics, collaboration networks, and keyword co‐occurrence.

**Results:**

Among 217 publications, research output increased 13‐fold (2005–2023), with Shock and Critical Care as leading journals. The United States, Japan, and Australia were top contributors, with strong international collaborations. Key themes included EG biomarkers, microcirculatory dysfunction, and therapeutic strategies. Emerging trends involved COVID‐19–associated EG injury and novel imaging techniques.

**Conclusion:**

This study maps the evolution of EG research in sepsis, highlighting exponential growth, key contributors, and thematic shifts. Future directions include validating EG biomarkers, developing targeted therapies, and enhancing global research equity.

## 1. Introduction

Sepsis continues to pose a significant global health challenge, characterized by high rates of morbidity and mortality [1]. Sepsis is a complex, dysregulated host response to infection that arises from dynamic interactions between invading pathogens and the host immune system. Although immune activation and pathogen burden initiate the septic process, downstream vascular and endothelial alterations—including glycocalyx injury—play a critical role in amplifying inflammation, microcirculatory dysfunction, and organ failure. Rather than acting in isolation, glycocalyx degradation reflects the integrated effects of pathogen‐derived factors, host immune activation, inflammatory mediators, and endothelial injury [2]. The endothelial glycocalyx is a delicate, carbohydrate‐rich layer composed of glycoproteins and proteoglycans lining the luminal surface of blood vessels [3]. It plays a crucial role in maintaining vascular homeostasis by regulating permeability, leukocyte adhesion, and coagulation [4]. During sepsis, the integrity of the endothelial glycocalyx is compromised, leading to increased vascular permeability, edema, and subsequent organ dysfunction [5].

Recent studies have highlighted the role of inflammatory mediators, such as interleukin‐6 (IL‐6), in driving endothelial glycocalyx degradation in both bacterial sepsis and COVID‐19 [6]. Elevated IL‐6 levels have been associated with reduced endothelial glycocalyx thickness, as measured by sublingual video‐microscopy, and increased circulating biomarkers indicative of glycocalyx damage [6]. In vitro experiments have demonstrated that IL‐6 can directly reduce endothelial glycocalyx height and coverage, effects that are mitigated by IL‐6 signaling inhibitors like tocilizumab and tofacitinib [7]. In clinical contexts, glycocalyx degradation reflects a systemic vascular phenomenon rather than a compartment‐specific process; therefore, the term “glycocalyx” is used herein to denote the whole‐body vascular glycocalyx unless otherwise specified, whereas “endothelial glycocalyx” is reserved for mechanistic or microscopic endothelial‐level investigations.

Therapeutic interventions aimed at preserving or restoring the endothelial glycocalyx have gained attention [8]. Therapeutic plasma exchange has been shown to reduce circulating levels of heparan sulfate, a component of the endothelial glycocalyx, and restore the balance between heparanase enzymes, thereby attenuating endothelial glycocalyx degradation in septic patients [9]. Additionally, the use of sulodexide, a heparan sulfate‐like compound resistant to degradation by heparanase, has demonstrated protective effects on the glycocalyx in sepsis models [10]. Advancements in imaging techniques, such as high‐resolution sublingual video‐microscopy, have facilitated real‐time assessment of the endothelial glycocalyx in septic patients, providing valuable insights into the extent of glycocalyx damage and its correlation with disease severity [11]. These technological developments have enhanced our understanding of the dynamic changes occurring in the microcirculation during sepsis [12]. The COVID‐19 pandemic has further underscored the importance of the endothelial glycocalyx in critical illness [13]. Patients with severe COVID‐19 exhibit significant endothelial glycocalyx degradation, similar to that observed in bacterial sepsis, suggesting shared pathophysiological mechanisms involving endothelial injury and inflammation [13].

Given the expanding body of research on the endothelial glycocalyx in sepsis, a comprehensive bibliometric analysis is warranted to systematically evaluate global research trends, identify key contributors, and map the evolution of scientific knowledge in this domain. Such an analysis can provide valuable insights into the trajectory of endothelial glycocalyx research, highlight emerging themes, and inform future investigative directions. This study is aimed at (A) quantifying the growth of research on the endothelial glycocalyx in sepsis from 2005 to 2025; (B) identifying leading authors, institutions, countries, and journals contributing to this field; (C) examining collaboration networks and thematic shifts over time; and (D) highlighting emerging trends and potential future research directions. By mapping research trends and collaborations, this bibliometric analysis seeks to provide a comprehensive overview of the current state of endothelial glycocalyx research in sepsis, thereby facilitating the identification of knowledge gaps and opportunities for interdisciplinary collaboration and translational research.

## 2. Materials and Methods

This bibliometric analysis systematically evaluates global research trends on the role of the endothelial glycocalyx in sepsis. The study employs quantitative and visualization techniques to analyze publication patterns, collaborations, and emerging themes.

### 2.1. Search Strategy and Data Collection

**Figure 1:**
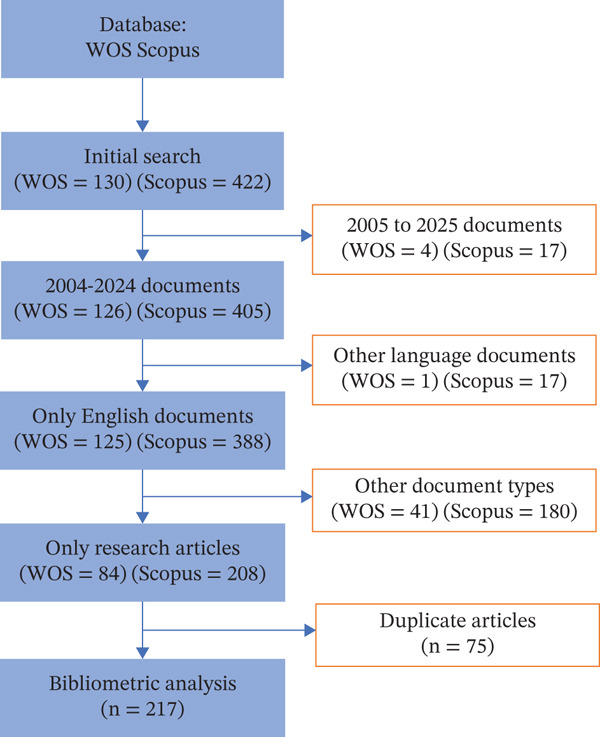
Flowchart for article selection in the bibliometric analysis of global research trends on endothelial glycocalyx in sepsis.

Data were extracted from the Web of Science Core Collection and Scopus, two widely recognized databases for high‐quality, peer‐reviewed literature. A combination of search terms related to sepsis and endothelial glycocalyx was applied using Boolean operators (Table 1). The search covered publications from January 2005 to May 2025 to capture evolving research trends over the past two decades. The inclusion criteria were limited to original research articles published in English. Exclusion criteria included review articles, editorials, letters, and publications in languages other than English (Figure 1). Duplicate records were identified and removed using a custom RStudio script (Table 2).

**Table 1:** Search strategy using Web of Science and Scopus databases (author keywords, title, or abstract) for the bibliometric analysis of global research trends on endothelial glycocalyx in sepsis.

Code	Queries
#1	“Sepsis” or “Bloodstream Infection” or “Bloodstream Infections” or “Infection, Bloodstream” or “Septicemia” or “Septicemias” or “Blood Poisoning” or “Blood Poisonings” or “Poisonings, Blood” or “Poisoning, Blood” or “Severe Sepsis” or “Sepsis, Severe” or “Pyemia” or “Pyemias” or “Pyaemia” or “Pyaemias” or “Pyohemia” or “Pyohemias” or “Shock, Septic” or “Septic Shock” or “Shock, Endotoxic” or “Endotoxin Shock” or “Endotoxin Shocks” or “Shock, Endotoxin” or “Shocks, Endotoxin” or “Shock, Toxic” or “Toxic Shock” or “Toxic Shock Syndrome” or “Shock Syndrome, Toxic” or “Toxic Shock Syndromes” or “Endotoxemia” or “Endotoxemias”
#2	“Endothelium” or “Endotheliums”
#3	“Glycocalyx” or “Cell Coat” or “Cell Coats” or “Coat, Cell” or “oats, Cell” or “Glycocalix” or “Syndecan‐1” or “Syndecan 1” or “CD138 Antigen” or “Antigen, CD138” or “Antigens, CD138” or “CD138 Antigens” or “Heparitin Sulfate” or “Sulfate, Heparitin” or “Heparan Sulfate” or “Sulfate, Heparan”
#3	#1, #2, and #3

**Table 2:** Script used to merge Web of Science and Scopus datasets and remove duplicates for bibliometric analysis.

Library (bibliometrix)
Library (openxlsx)
#Importing the Web of Science dataset web_data <‐ convert2df (“abs.txt”)
#Importing the Scopus dataset scopus_data <‐ convert2df (“abs.bib”, dbsource = ^“^scopus^”^, format = ^“^bibtex^”^)
#Merging both datasets while removing duplicates combined <‐ mergeDbSources (web_data, scopus_data, remove. Duplicated = true)
#Exporting the combined dataset to an Excel file write.xlsx (combined, “combinedabs.xlsx”)

### 2.2. Analysis Method

Bibliometric analysis was performed using the Bibliometrix R package, a powerful tool for science mapping and quantitative research evaluation. The analysis examined annual scientific production, identifying the most productive countries, institutions, and authors contributing to research on endothelial glycocalyx in sepsis. Citation data, including total citations and journal impact factors, were used to assess the scientific influence of the publications. Collaboration patterns were explored through coauthorship network analysis, with betweenness centrality applied to determine key nodes that serve as bridges within the scientific community. Thematic trends were analyzed using keyword co‐occurrence to uncover major research areas and emerging topics in the field. Visualization techniques such as network maps were used to illustrate coauthorship and institutional collaborations, whereas thematic maps and word clouds highlighted keyword clusters and research hotspots. All analyses and visualizations were conducted in RStudio (Version 2024.12.1 Build 563) using functions available within the Bibliometrix and Biblioshiny packages.

Collaboration networks were visualized using the Biblioshiny interface of the Bibliometrix R package. Coauthorship and institutional collaboration maps were generated based on network analysis of authorship metadata, where node size represents publication output and edge thickness reflects collaboration strength. To enhance readability, figures were exported at high resolution with optimized layout parameters, allowing clearer visualization of major collaborative clusters.

## 3. Results

### 3.1. Comprehensive Overview of the Manuscripts

A comprehensive analysis was conducted on 217 relevant publications derived from 133 distinct sources. These studies were authored by 1545 researchers, yielding an average of 40.22 citations per publication over the last two decades. Key insights into the endothelial glycocalyx in the context of sepsis are summarized in Table 3, which highlights the most frequently cited articles from the past 10 years. A total of 489 unique author‐provided keywords were identified. Collaborative authorship accounted for 11.06% of the publications.

**Table 3:** Top 10 most cited documents in the bibliometric analysis of global research trends on endothelial glycocalyx in sepsis (2005–2025).

Rank	First author, year	Title of the document	Journal name	Total citations	Average annual citations	DOI
1	Schmidt (2012) [14]	The Pulmonary Endothelial Glycocalyx Regulates Neutrophil Adhesion and Lung Injury During Experimental Sepsis	Nat Med	667	44.47	10.1038/nm.2843
2	Schouten (2008) [15]	Inflammation, Endothelium, and Coagulation in Sepsis	J Leukocyte Biol	491	25.84	10.1189/jlb.0607373
3	Steppan (2011)[16]	Sepsis and Major Abdominal Surgery Lead to Flaking of the Endothelial Glycocalix	J Surg Res	220	39.45	10.1016/j.jss.2009.04.034
4	Chappell (2009) [17]	TNF‐*α* Induced Shedding of the Endothelial Glycocalyx Is Prevented by Hydrocortisone and Antithrombin	Basic Res Cardiol	212	28.58	10.1007/s00395-008-0749-5
5	Nelson (2008)[18]	Increased Levels of Glycosaminoglycans During Septic Shock: Relation to Mortality and the Antibacterial Actions of Plasma	Shock	210	13.75	10.1097/SHK.0b013e3181777da3
6	Marechal (2008)[19]	Endothelial Glycocalyx Damage During Endotoxemia Coincides With Microcirculatory Dysfunction and Vascular Oxidative Stress	Shock	182	11.78	10.1097/SHK.0b013e318157e926
7	Han (2016) [20]	Amelioration of Sepsis by TIE2 Activation–Induced Vascular Protection	Sci Transl Med	169	11.05	10.1126/scitranslmed.aad9260
8	Xu (2014)[21]	TNF‐Mediated Damage to Glomerular Endothelium Is an Important Determinant of Acute Kidney Injury in sepsis	Kidney Int	163	9.58	10.1038/ki.2013.286
9	Hippensteel (2019)[22]	Intravenous Fluid Resuscitation Is Associated With Septic Endothelial Glycocalyx Degradation	Crit Care	148	30	10.1186/s13054-019-2534-2
10	Wiesinger (2013)[23]	Nanomechanics of the Endothelial Glycocalyx in Experimental Sepsis	Plos One	130	15.36	10.1371/journal.pone.0080905

To mitigate the influence of publication age on citation‐based rankings, we additionally calculated the average annual citation rate for the top‐cited documents (Table 3). Although earlier landmark studies naturally accumulated higher total citations, several more recent publications demonstrated comparatively high average annual citation rates indicating strong field‐normalized impact despite shorter citation windows. These complementary metrics provide a time‐adjusted perspective on scholarly influence and better reflect the contemporary relevance of recent contributions to “glycocalyx” research in sepsis.

### 3.2. Evolution of Publication and Citation Metrics

An overall upward trajectory in research publications related to the endothelial glycocalyx in sepsis is evident (Figure 2a,b). From 2005 to 2023, the yearly output expanded significantly, increasing over 13 times—from just three papers in 2005 to a peak of 39 in 2023. This growth reflects a heightened level of academic interest and a broader recognition of the topic′s relevance within sepsis research.

Figure 2The annual global trends in (a) the number of publications, (b) citation frequency, and (c) the distribution of relevant literature based on Bradford′s law, which pinpointed 10 core journals that have consistently published on the topic of endothelial glycocalyx in sepsis between 2005 and 2025.

**Figure:**
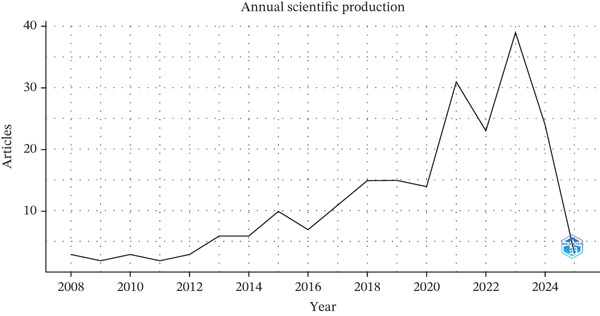
(a)

**Figure:**
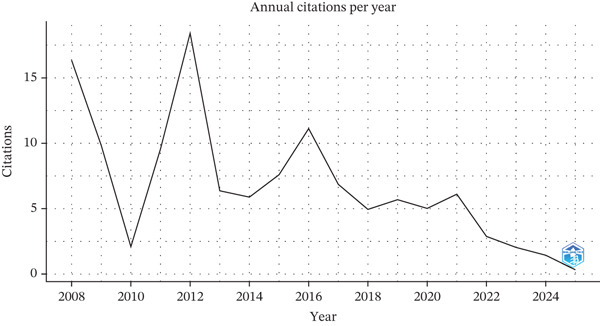
(b)

**Figure:**
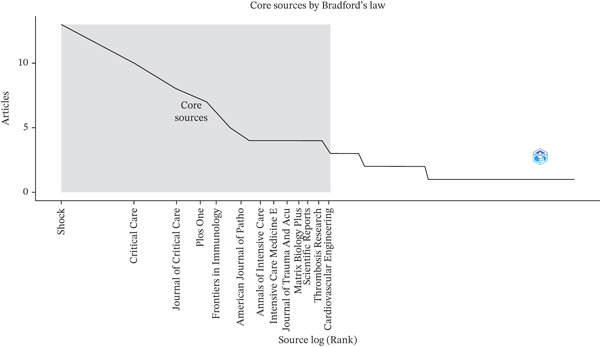
(c)

Although the volume of publications has grown, the average citation count per article has shown a downward trend, dropping from 293.33 in 2005 to only 0.33 in 2025. Average citations per article were lower in the most recent years. A corresponding reduction in the annual mean citation rate was also observed, decreasing from 14.4 to 0.3 during the same time span. Still, the steady increase in publication numbers illustrates the enduring commitment of the research community to this area of investigation.

By applying Bradford′s law, 10 core journals were identified as the principal sources for research on endothelial glycocalyx in sepsis (Figure 2c). Shock emerged as the most prolific journal with 13 articles, followed by Critical Care with 10, Journal of Critical Care with eight, Plos One with seven, and Frontiers in Immunology with five. Other notable journals include The American Journal of Pathology, Annals of Intensive Care, Intensive Care Medicine Experimental, Journal of Trauma and Acute Care Surgery, and Matrix Biology Plus—each contributing four publications.

These journals serve as the foundational channels for publishing in this niche, indicating a concentrated scholarly engagement. Their recurring presence as publication venues underlines their important role in facilitating the dissemination of findings and fostering the advancement of knowledge in this specialized field.

### 3.3. Leading Authors, Institutions, Countries, and Collaboration Networks

Between 2005 and 2025, several institutions emerged as key contributors to research on the endothelial glycocalyx in sepsis. The most prolific organizations included Copenhagen University Hospital and the University of Copenhagen, each producing 30 publications. They were followed by the University of Alabama at Birmingham (21 publications), Gifu University [24], the University of Amsterdam [25], Rigshospitalet [26], the University of Münster [27], the University of California system [14], the University of Western Australia [13], and University Hospital Münster [11] (Figure 3a). This distribution indicates a wide global engagement, with notable representation from Europe, North America, Asia, and Oceania, highlighting the international and interdisciplinary nature of this research field.

Figure 3Summary of leading contributors and emerging patterns in research on endothelial glycocalyx in sepsis from 2005 to 2025. (a) A collaboration map showcasing the top authors, institutions, and countries actively engaged in this field; (b) timeline illustrating the publication activity of the 10 most impactful authors over the 2005–2025 period; and (c) a three‐field plot demonstrating the relationships among cited references (CR), contributing authors (AU), and frequently used author keywords (DE) throughout the study period.

**Figure:**
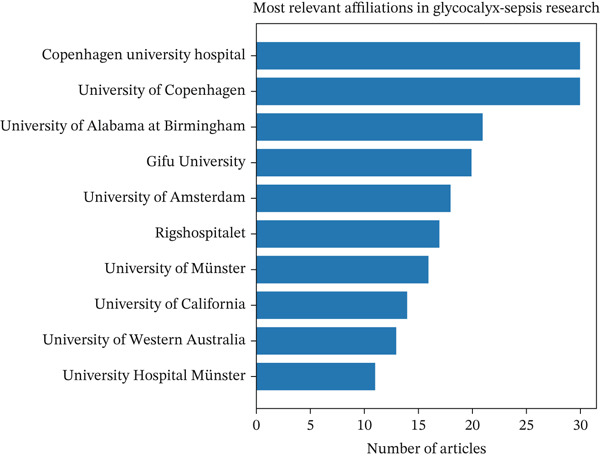
(a)

**Figure:**
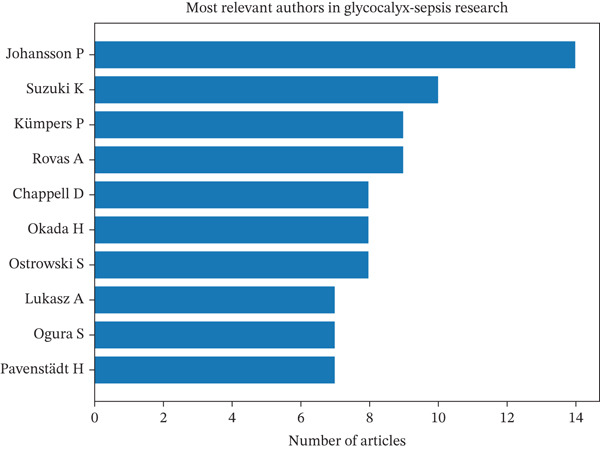
(b)

**Figure:**
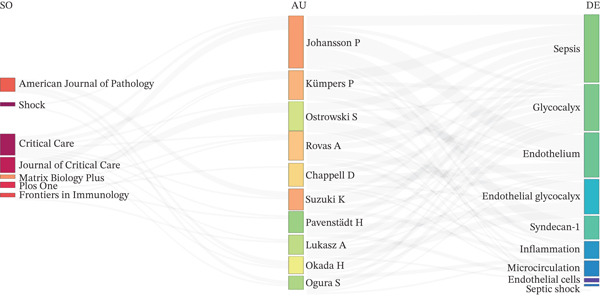
(c)

Regarding individual author contributions, the most productive researchers over the two‐decade span included Johansson (14 publications), Suzuki [10], Kümpers [9], Rovas [9], Chappell [8], Okada [8], Ostrowski [8], Lukasz [7], Ogura [7], and Pavenstädt [7] (Figure 3b). Many of these authors are affiliated with the aforementioned top institutions, suggesting concentrated efforts within certain academic centers that lead this field.

The three‐field plot (Figure 3c) presents a visual overview of connections among the most frequently cited references, prolific authors, and recurring author keywords. This plot underscores the collaborative framework of the field, linking researchers to specific thematic foci and prominent publishing outlets.

Core journals such as Shock, Critical Care, and The American Journal of Pathology were among the main publication venues in this domain. Leading authors were frequently linked with recurring keywords such as “glycocalyx,” “syndecan‐1,” and “septic shock” in the three‐field plot (Figure 3c).

Keyword co‐occurrence analysis further reveals a strong focus on pathophysiological processes such as endothelial dysfunction and inflammation. The frequent association of the biomarker “syndecan‐1” with clinical terms such as “septic shock” highlights its diagnostic and prognostic relevance within the research landscape on endothelial injury in sepsis.

The country collaboration map (Figure 4a) reveals the extent of international cooperation in endothelial glycocalyx research related to sepsis. The United States emerged as the dominant contributor, with 113 publications, followed by Japan (65) and Australia (59). Notably, the United States also exhibited the most extensive collaborative network, with strong ties to Denmark (six collaborations), Japan [3], and multiple European nations, reflecting its integral role in advancing global endothelial glycocalyx–sepsis research.

Figure 4Global collaboration patterns and keyword trends in glycocalyx research in sepsis (2005–2025). (a) International country collaboration network with country labels displayed, node size represents publication output and link thickness reflects collaboration strength. (b) TreeMap of the most frequent author keywords. (c) Temporal evolution of major research keywords in glycocalyx‐related sepsis research (2005–2025), illustrating a shift from early pathophysiological studies toward biomarker identification, microcirculatory dysfunction, and therapeutic strategies in recent years.

**Figure:**
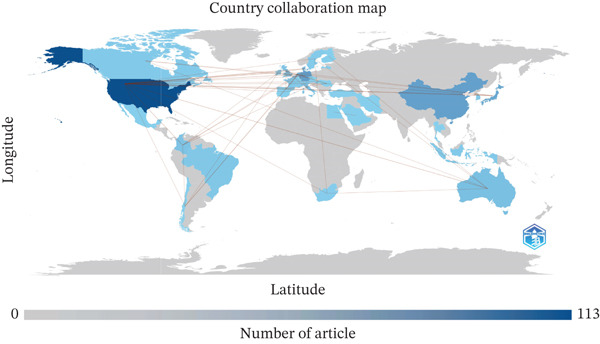
(a)

**Figure:**
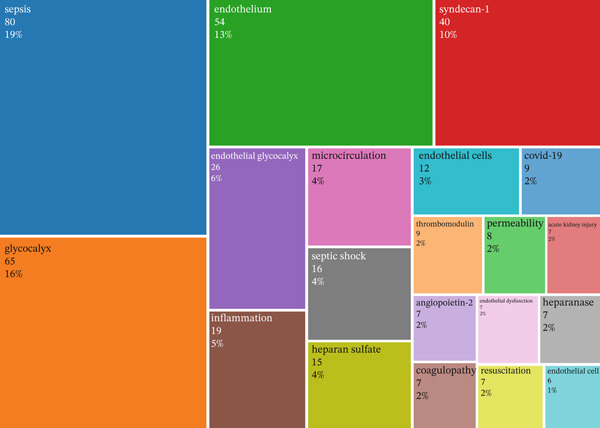
(b)

**Figure:**
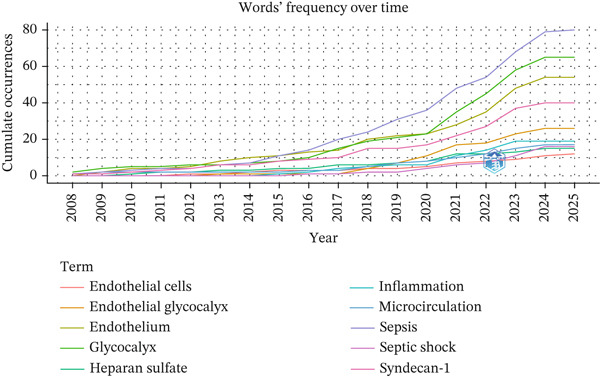
(c)

### 3.4. Co‐Occurrence, Focal Points, and Evolving Keywords

The TreeMap visualization (Figure 4b) illustrates the relative prominence and evolving relevance of core terms in the field of endothelial glycocalyx research related to sepsis. To avoid bias introduced by predefined search terms, keywords directly reflecting the search strategy were excluded from thematic interpretation. After this adjustment, keyword co‐occurrence analysis revealed several prominent research hotspots. High‐frequency mechanistic and clinical terms included “inflammation,” “microcirculation,” “vascular permeability,” and “endothelial dysfunction,” indicating a strong focus on immune‐mediated vascular injury and impaired tissue perfusion. In addition, biomarkers such as “syndecan‐1” and “heparan sulfate” emerged as central nodes, highlighting their growing importance in assessing glycocalyx injury and disease severity in sepsis.

Complementing this, the longitudinal keyword trend analysis (Figure 4c) indicates increasing attention over time to clinically and mechanistically informative terms, including “syndecan‐1,” “heparan sulfate,” “microcirculation,” “vascular permeability,” and “inflammation.” These trends suggest a growing emphasis on biomarker development and microvascular dysfunction alongside endothelial injury mechanisms.

The temporal evolution of research themes, illustrated in Figure 4c, demonstrates a clear shift in focus over time. Early publications (pre‐2015) predominantly addressed the general pathophysiology of sepsis and endothelial dysfunction. In contrast, more recent studies increasingly emphasize clinically actionable topics, including glycocalyx‐related biomarkers, microcirculatory dysfunction, vascular permeability, and therapeutic strategies aimed at endothelial protection. This thematic progression reflects a transition from descriptive mechanistic research toward translational and clinically oriented investigations.

## 4. Discussion

The analysis of 217 publications from 133 sources, involving 1545 researchers, underscores the growing scientific interest in the endothelial glycocalyx in sepsis. The average citation rate of 40.22 per publication indicates substantial academic impact, particularly for seminal works such as Schmidt et al. [14] and Chappell et al. [17], which have shaped current understanding of glycocalyx degradation in sepsis. The high citation counts of these foundational studies reflect their role in establishing key mechanistic insights, such as neutrophil adhesion [14] and TNF‐*α*–mediated shedding [17].

Interpretation of citation‐based influence should consider temporal bias, as older publications inherently accrue more citations over time. By incorporating average annual citations, this analysis demonstrates that several recent studies exert substantial normalized influence within the field despite lower absolute citation counts. These findings emphasize the importance of using normalized bibliometric indicators alongside total citations to more accurately capture scientific impact across publication years.

The identification of 489 unique keywords highlights the thematic diversity in this field, spanning molecular mechanisms, clinical outcomes, and therapeutic strategies. The relatively low rate of collaborative authorship (11.06%) suggests that although interdisciplinary cooperation exists, there remains room for expanded partnerships, particularly between basic scientists and clinical researchers. This aligns with broader trends in sepsis research, where translational gaps persist between mechanistic discoveries and clinical applications [27]. Thus, fostering larger, multicenter collaborations could accelerate the translation of glycocalyx research into therapeutic innovations.

The exponential growth in publications—from three in 2005 to 39 in 2023—demonstrates the rising recognition of the endothelial glycocalyx′s role in sepsis pathophysiology. This trend mirrors advancements in vascular biology and critical care medicine, particularly after the sepsis‐3 definitions [26] emphasized endothelial dysfunction as a key feature of sepsis. However, the decline in average citations per paper (293.33 in 2005 vs. 0.33 in 2025) is expected, as newer studies require time to accumulate citations. This pattern is consistent with bibliometric analyses in other biomedical fields, where early foundational works dominate citations before newer contributions gain traction [25].

Bradford′s law identified Shock and Critical Care as core journals, reflecting their focus on sepsis and microcirculatory dysfunction. The prominence of these journals suggests that endothelial glycocalyx research is primarily disseminated within critical care and shock research communities, with fewer publications in broader vascular biology journals. This indicates a potential opportunity for cross‐disciplinary engagement with cardiovascular and glycobiology journals to widen the impact of endothelial glycocalyx research.

The dominance of institutions like the University of Copenhagen and the University of Alabama at Birmingham highlights their important role in advancing endothelial glycocalyx–sepsis research. These centers have produced high‐impact studies on glycocalyx biomarkers and therapeutic targets [20]. The concentration of prolific authors within these institutions suggests that specialized research hubs drive innovation in this niche.

Geographically, the United States, Japan, and Australia lead in output, with the United States serving as the integral node in international collaborations. This aligns with global sepsis research trends, where high‐income countries dominate due to greater funding and infrastructure [24]. However, the limited representation from low‐ and middle‐income countries (LMICs)—where sepsis burden is highest—points to a research disparity. Increasing investment in LMIC‐based sepsis research could provide insights into region‐specific glycocalyx dynamics and therapeutic needs.

To improve interpretability, country labels were added directly to the international collaboration network (Figure 4a). The United States occupied a central position within the global collaboration structure, maintaining strong bilateral links with Denmark, Japan, Australia, and several European countries. In contrast, collaborations among Asian and European countries were more regionally clustered. To further contextualize these patterns, a summary of the dominant thematic focus of international collaborations by country is presented in Table 4, highlighting how national research efforts converge on specific mechanistic and clinical aspects of glycocalyx injury in sepsis.

**Table 4:** International collaboration patterns and dominant research focus areas in glycocalyx research related to sepsis.

Country	Major international collaborators	Dominant research focus areas
United States	Denmark, Japan, United Kingdom, Germany, and Australia	Inflammation, microcirculation, and vascular permeability
Denmark	United States, Germany, and Netherlands	Glycocalyx structure and endothelial barrier function
Japan	United States, China, and South Korea	Biomarkers, syndecan‐1, and experimental sepsis models
Australia	United States and United Kingdom	Microcirculatory dysfunction and translational studies
Germany	United States, Denmark, and Netherlands	Endothelial dysfunction and heparan sulfate metabolism
Netherlands	Denmark, Germany, and United States	Vascular permeability and microvascular imaging
United Kingdom	United States and Australia	Clinical sepsis phenotyping and inflammation

Keyword analysis reveals a shift from broad sepsis investigations (pre‐2015) to focused studies on glycocalyx‐specific mechanisms (post‐2015). The rising prominence of terms like syndecan‐1 and heparan sulfate reflects growing interest in glycocalyx degradation biomarkers and their clinical utility [28]. Meanwhile, sustained attention to microcirculation and inflammation underscores the endothelial glycocalyx′s integral role in sepsis‐induced vascular dysfunction

### 4.1. Recent Developments (2020–2025)

Recent studies have expanded the scope of glycocalyx research in sepsis by integrating emerging clinical contexts, therapeutic concepts, and advanced imaging techniques. Notably, the COVID‐19 pandemic has reinforced the relevance of glycocalyx injury in critical illness, as severe COVID‐19 shares key pathophysiological features with bacterial sepsis, including systemic inflammation, endothelial dysfunction, and glycocalyx degradation [6, 13]. These observations have stimulated comparative investigations into shared mechanisms of vascular injury and potential therapeutic targets across infectious syndromes.

In parallel, increasing attention has been directed toward glycocalyx‐protective strategies, including plasma‐based interventions, endothelial barrier–stabilizing agents, and approaches targeting heparanase‐mediated shedding [10, 20, 27]. Advances in imaging and analytical techniques, such as sublingual intravital microscopy and ultrastructural visualization of glycocalyx dynamics, have further enabled in vivo assessment of glycocalyx integrity and its association with microcirculatory dysfunction and clinical outcomes [11]. Collectively, these developments indicate a shift from descriptive characterization toward translational and clinically oriented research, positioning the glycocalyx as both a biomarker and a potential therapeutic target in sepsis management.

### 4.2. Future Research Directions and Gaps

Although the literature on glycocalyx injury in sepsis has expanded rapidly, several high‐priority directions remain. First, prospective, multicenter clinical studies should validate circulating glycocalyx biomarkers—particularly syndecan‐1, heparan sulfate, and hyaluronan—using standardized sampling time‐points and clinically meaningful endpoints (mortality, organ dysfunction trajectories, and microcirculatory impairment). Second, future work should harmonize bedside assessment protocols for glycocalyx integrity and microcirculation to improve comparability across cohorts and facilitate meta‐research. Third, translational studies should prioritize mechanism‐guided interventions and define patient subphenotypes most likely to benefit, linking inflammatory pathways, endothelial permeability, and microcirculatory failure to measurable glycocalyx injury. These interpretation‐focused points are discussed here rather than in the Results to maintain a descriptive presentation of bibliometric findings.

From a therapeutic perspective, randomized or well‐designed pragmatic trials are needed to test glycocalyx‐protective strategies with biomarker‐guided enrichment designs. In parallel, preclinical studies should use clinically relevant models and report reproducible glycocalyx outcomes using consistent staining/imaging and quantification methods. Finally, the bibliometric landscape reveals geographic imbalance: Regions with high sepsis burden contribute relatively fewer studies, highlighting the need for capacity building, shared protocols, and international consortia that include low‐ and middle‐income settings to improve global relevance and equity.

### 4.3. Therapeutic and Clinical Implications of Glycocalyx Research

Growing evidence indicates that glycocalyx injury is not only a marker of disease severity but also a potential therapeutic target in sepsis. Research in this field has contributed to improved diagnostic stratification, as circulating glycocalyx components such as syndecan‐1 and heparan sulfate have been associated with endothelial damage, microcirculatory impairment, and adverse clinical outcomes. These biomarkers offer opportunities for early risk stratification and may support biomarker‐guided therapeutic decision‐making in critically ill patients.

From a therapeutic perspective, glycocalyx‐focused research has informed the development and evaluation of glycocalyx‐preserving strategies, including plasma‐based interventions, modulation of heparanase activity, anti‐inflammatory approaches, and endothelial barrier–stabilizing agents. Experimental and early clinical studies suggest that preserving glycocalyx integrity may attenuate vascular permeability, improve microcirculatory perfusion, and reduce downstream organ dysfunction. However, translation into routine clinical practice remains limited, highlighting the need for well‐designed clinical trials that integrate glycocalyx biomarkers as inclusion criteria or response indicators. Addressing these translational gaps will be essential to transform glycocalyx research from a predominantly descriptive domain into a clinically actionable component of sepsis management.

## 5. Conclusion

This bibliometric analysis demonstrates a clear temporal evolution in research on glycocalyx involvement in sepsis. As illustrated by the keyword timeline analysis, early studies primarily focused on elucidating the pathophysiological role of endothelial injury and vascular dysfunction in sepsis, whereas more recent research has increasingly shifted toward clinically oriented themes, including biomarker identification, microcirculatory assessment, and the development of targeted therapeutic strategies. This progression reflects the maturation of the field from descriptive mechanistic investigations toward translational and patient‐relevant applications.

Despite this advancement, several challenges remain. A major limitation is the persistent translational gap, as many mechanistic insights derived from experimental and preclinical studies have yet to be systematically validated in well‐designed clinical trials. In addition, the bibliometric landscape reveals a marked geographic imbalance in research output, with LMICs—despite bearing a substantial proportion of the global sepsis burden—remaining underrepresented. Furthermore, limited interdisciplinary integration may constrain innovation, underscoring the need for closer collaboration with fields such as glycobiology, bioengineering, and microvascular imaging.

Future research should therefore prioritize (1) multicenter clinical validation of glycocalyx injury biomarkers using standardized sampling time‐points and clinically meaningful endpoints, (2) harmonization of protocols for microcirculatory and glycocalyx imaging to improve cross‐study comparability, (3) biomarker‐guided interventional trials evaluating glycocalyx‐stabilizing and endothelial‐protective therapies, and (4) strengthened global research collaborations to reduce geographic disparities and enhance the generalizability of evidence. Addressing these priorities will accelerate the translation of glycocalyx‐related research from descriptive insights to clinically actionable diagnostics and therapeutic strategies in sepsis care.

## Author Contributions

Kamila R. Daniyarova, Zhanslu N. Sarkulova, and Amin Tamadon contributed equally to the conception and design of the study, systematic literature search, data extraction, and drafting of the manuscript. Ainur B. Tokshilykova and Botagoz M. Kalieva participated in data collection, quality assessment of included studies, and assisted in statistical analysis. Marat N. Sarkulov and Nadiar M. Mussin contributed to data interpretation, critical revision of the manuscript for important intellectual content, and provided clinical expertise in sepsis management. Ramazon Safarzoda Sharoffidin supervised the overall project, contributed to study conceptualization and methodology, resolved discrepancies during data extraction, and served as the corresponding author. Kamila R. Daniyarova, Zhanslu N. Sarkulova, and Amin Tamadon have the same contributions as the first coauthor.

## Funding

No funding was received for this manuscript.

## Disclosure

All authors have read and approved the final version of the manuscript.

## Ethics Statement

The authors have nothing to report.

## Consent

The authors have nothing to report.

## Conflicts of Interest

The authors declare no conflicts of interest.

## Data Availability

The data presented in this study are available on request from the corresponding author.
